# Advancement of PD Is Reflected by White Matter Changes in Olfactory Areas: A Pilot Study

**DOI:** 10.3390/medicina57111183

**Published:** 2021-11-01

**Authors:** Thomas Hummel, Antje Haehner, Divesh Thaploo, Charalampos Georgiopoulos, Björn Falkenburger, Katherine Whitcroft

**Affiliations:** 1 Smell & Taste Clinic, Department of Otorhinolaryngology, TU Dresden, 01307 Dresden, Germany; thomas.hummel@tu-dresden.de (T.H.); Antje.Haehner@uniklinikum-dresden.de (A.H.); charalampos.georgiopoulos@liu.se (C.G.); 2 Department of Radiology in Linköping, Linköping University, 58183 Linköping, Sweden; 3 Department of Health, Medicine and Caring Sciences, Linköping University, 58183 Linköping, Sweden; 4 Department of Neurology, TU Dresden, 01307 Dresden, Germany; bjoern.falkenburger@ukdd.de; 5 UCL Ear Institute, UCL, 332 Grays Inn Rd., London WC1X 8EE, UK; k.whitcroft@gmail.com

**Keywords:** Parkinson’s disease, diffusion tensor imaging, left posterior piriform cortex, orbitofrontal cortex

## Abstract

Loss of sense of smell is a well-known non-motor symptom of Parkinson’s disease (PD). Here, we present insight into the association between PD advancement and equivalents of smell loss in olfactory-eloquent brain areas, such as the posterior cortex and orbitofrontal cortex. Twelve PD patients in different Hoehn and Yahr stages and 12 healthy normosmic individuals were examined with diffusion tensor imaging. Tract-based spatial statistics were used to analyze microstructural changes in white matter adjacent to the bilateral posterior and orbitofrontal cortex. Axial diffusivity, mean diffusivity, and radial diffusivity were significantly higher in olfactory ROIs in advanced PD patients. The results of this preliminary study indicate that PD advancement is associated with progressive neurodegeneration in olfactory-related brain areas.

## 1. Introduction

Parkinson’s disease (PD) is characterized by a variety of symptoms ranging from motor to mental disability. Olfactory loss is widely acknowledged as one of the major non-motor symptoms of the disease which precedes the occurrence of clinical motor symptoms [1]. Few studies have reported an association between disease severity and smell loss [2,3].

Diffusion tensor imaging (DTI) has been previously used to assess white matter changes in olfactory-eloquent brain areas [4,5]. Voxel-based analyses of DTI data of PD patients showed reduced fiber integrity as indicated by fractional anisotropy (FA) values [6]. In one study, FA in olfactory cortex areas correlated with scores from an odor identification test [5]. However, other studies did not observe such associations. 

Due to the inconsistent results of previous studies, it is still unclear whether disease advancement, in terms of both severity and duration, is associated with progressive degeneration in olfactory brain areas. The aim of this study was to re-investigate the correlation between severity and duration of PD and white matter changes in olfactory brain areas in PD patients compared to healthy, normosmic individuals. 

## 2. Materials and Methods

Twelve PD patients (mean age = 62.5 ± 7.39 years; 8 men and 4 women; mean disease duration = 6.6 ± 2.7 years) at Hoehn and Yahr stages 1–3 were recruited from the Department of Neurology at the TU Dresden, as well as from the local Parkinson support group with informed consent, abiding by the local ethics committee of TU Dresden with approval number 262082010 and approval date 24 April 2014. The Unified Parkinson’s Disease Rating Scale (UPDRS) ranged from 7 to 49 (mean = 23.6 ± 13.3; ON-rating). Exclusion criteria were head injury, other CNS-related diseases, and sinonasal diseases. Twelve healthy control subjects (CONTROL; mean age = 57.3 ± 8.4; 11 men and 1 woman) with a normal sense of smell were recruited from the general population via local advertisements.

Olfactory testing was performed using the “Sniffin’ Sticks” test kit involving extensive tests for odor threshold, discrimination, and identification. The sum of the scores from the three subtests resulted in the TDI-score (threshold, discrimination, identification), which allowed for the categorization of participants as anosmic, hyposmic, or normosmic [7].

A 3 T MRI scanner (Verio; Siemens Healthineers, Erlangen, Germany) equipped with an eight-channel receiver head-coil was used for image data acquisition. DTI was acquired through 2D fast-spin echo planar imaging with the following specifications; TR = 71 ms; TE = 6 ms; slice thickness = 2 mm; FoV = 110 × 110; repetitions = 1; and flip angle = 180°. Diffusion scans were acquired at b = 0 and b = 800 with number of diffusion directions = 20. Single-subject diffusion maps were processed using tract-based spatial statistics (TBSS), which is part of the FSL software from the FMRIB software library (http://www.fmrib.ox.ac.uk/fsl) accessed on 20 March 2020. Region of interest (ROI) analysis was performed on the TBSS-normalized data, focusing on olfactory brain regions, namely the posterior and anterior posterior cortex (PC) and orbitofrontal cortex (OFC) bilaterally. The masks of these ROIs were designed and adapted from Seubert et al. [8] using the FSLeyes edit mode, where we overlayed the ROIs and established a threshold in order to ensure only white matter areas were included for the analysis. All analyses were carried out under the supervision of an expert neuroradiologist.

There were no significant differences between PD patients and healthy controls in terms of age (*t*-test, *p* = 0.08) or gender (chi-square, *p* = 0.225). We ran a partial correlation analysis among FA, MD, AD, and RD values and ROIs for PD patients, controlling for age. The level of significance was set at *p* < 0.05. Statistical analyses were performed using SPSS v25.

## 3. Results

**Figure 1:**
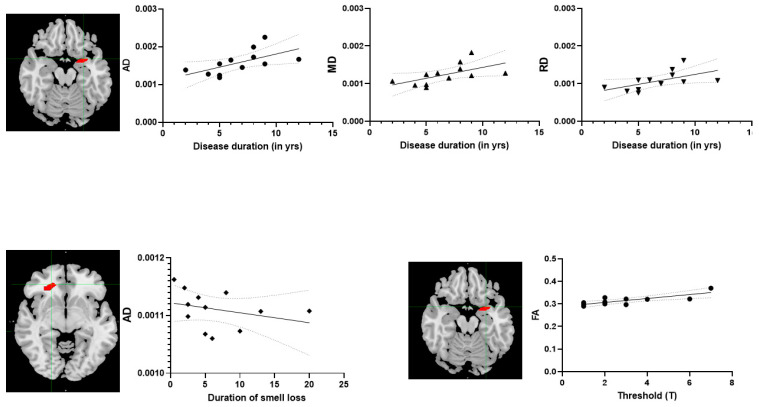
Partial correlation results. The **top row** represents the positive correlation with axial diffusivity (AD), mean diffusivity (MD), and radial diffusivity (RD) in the left posterior piriform cortex and regarding disease duration. The **bottom-row left** side shows a negative correlation with AD in the right orbitofrontal cortex and regarding the duration of smell loss. The **bottom-row right** side shows a positive correlation with fractional anisotropy (FA) in the left posterior piriform cortex and regarding threshold scores (T). ●, ▲ and ▼ in the **top row** represents the AD, MD and RD values of PD patients in left posterior piriform cortex and ♦, ● in the **bottom row** represents AD and FA values in right orbitofrontal cortex in PD patients.

Study demographics are shown in Table 1. The mean olfactory function (TDI score) of the PD group was significantly lower compared to the control group (*p* < 0.001). No correlation was found between the general TDI score and any of the DTI scalars across the groups. However, specific threshold scores were positively correlated with FA values in the left posterior piriform cortex (r = 0.82, *p* < 0.01) in the PD group. Partial correlation analysis, controlling for age, revealed that disease duration was positively correlated with AD (r = 0.71, *p* = 0.03), MD (r = 0.72, *p* = 0.02), and RD (r = 0.72, *p* = 0.02) values in the left posterior piriform cortex in the PD group. The duration of smell loss was negatively correlated with AD values in the right OFC (r = −0.71, *p* = 0.01). The disease severity in terms of the UPDRS score was positively correlated with AD (r = 0.88, *p* < 0.001), MD (r = 0.68, *p* = 0.015), and RD (r = 0.73, *p* = 0.006), and disease severity in terms of the Hoehn and Yahr score was positively correlated with AD (r = 0.87, *p* < 0.001), MD (r = 0.84, *p* = 0.001), and RD (r = 0.86, *p* < 0.001) in the right posterior piriform cortex. There was no correlation regarding OFC and UPDRS or Hoehn and Yahr. The correlation-based analysis is graphically represented in Figure 1.

## 4. Discussion

The aim of this study was to re-investigate whether there is an association between PD advancement and changes in olfactory brain areas. Three out of four DTI scalars, namely AD, MD, and RD in the left posterior piriform cortex, were found to be positively correlated with disease duration. This would support the results of a previous study suggesting that advanced stages of PD and increased severity of motor symptoms correlate with the degree of olfactory dysfunction [4]. The positive correlation between FA in the left posterior piriform cortex and the threshold score is in line with the notion that odor thresholds decline as a consequence of neurodegenerative progresses. The lack of a correlation for odor discrimination and identification scores can be explained by the idea that thresholds reflect aspects of olfactory processing related to the olfactory epithelium system, while odor discrimination and identification are more related to cognitive processes.

With the duration of smell loss, a negative correlation was seen with AD in OFC, indicating less axonal degeneration as the disease progresses. Since the OFC is a secondary olfactory area participating in numerous other functions, we speculate that OFC becomes less associated with olfaction in later stages of PD. So far, only one study has extensively examined olfactory areas in PD patients and included all four DTI scalars [9], and the authors of that study found changes in OFC and also in the entorhinal cortex in terms of AD values. However, no study to date has focused on the progression of PD in terms of DTI measures. The findings of the present study suggest that disease advancement is associated with local diffusion changes in olfactory brain areas rather than with demyelination or impaired axonal density. As the disease progresses, both in time and severity, the left posterior piriform cortex shows increased diffusion in all directions, which could be attributed to decreased cellularity due to degeneration [10]. This pilot study found a positive correlation between PD advancement and white matter areas surrounding the piriform cortex, suggesting that structural changes in olfactory areas become more prominent with disease advancement and could be regarded as a parameter for the progression of the disease. Although we had a small sample size, the results from our study are in line with the previous literature reporting changes in DTI measures in PD patients but not within olfactory areas. Future research should investigate how these measures respond when a larger sample size is taken into consideration. The strength of this study lies in the use of DTI measures for understanding the local changes with olfactory areas in PD. Although the small sample size is a weakness, it is important to note that we clearly saw changes in the olfaction-related areas. One limitation that could be easily overcome in future studies could be the use of better imaging given current advancements in diffusion imaging.

## 5. Conclusions

Smell loss predates motor loss in Parkinson’s disease; however, it has not yet been quantified how early. Through this study, we could demonstrate that there is a correlation between duration and severity of disease, and imaging markers such as AD, MD, and RD. Further investigations should be based on DTI markers to assess how smell loss progresses in PD.

## Figures and Tables

**Table 1:** Demographic details of the study population.

Groups	Age (in Years; Mean ± SD)	TDI (Mean ± SD)	Sex (m = Male; f = Female)	Duration of Smell Loss in Years (Mean ± SD)	UPDRS (Mean ± SD)	Hoehn and Yahr (Mean ± SD)	Threshold Score (Mean ± SD)	Disease Duration in Years (Mean ± SD)
PD	63.7 ± 4.4	17.5 ± 7.7	m = 8, f = 4	6.5 ± 5.5	23.7 ± 13.9	2.25 ± 0.58	2.9 ± 2.0	6.7 ± 2.7
CONTROL	57.3 ± 8.4	34.4 ± 3.0	m = 11, f = 1	Not applicable	Not applicable	Not applicable	7.4 ± 1.4	Not applicable

Abbreviations: PD = Parkinson’s disease; CONTROL = normosmics; TDI = total olfactory score combining odor threshold, odor discrimination, and odor identification; UPDRS = Unified Parkinson’s Disease Rating Scale; and H&Y = Hoehn and Yahr scale.
